# Prevalence of Subthreshold Depression Among Constipation-Predominant Irritable Bowel Syndrome Patients

**DOI:** 10.3389/fpsyg.2020.01936

**Published:** 2020-08-06

**Authors:** Norfilza Mohd Mokhtar, Mohd Fyzal Bahrudin, Nazierah Abd Ghani, Rafiz Abdul Rani, Raja Affendi Raja Ali

**Affiliations:** ^1^GUT Research Group, Faculty of Medicine, Universiti Kebangsaan Malaysia, Kuala Lumpur, Malaysia; ^2^Department of Physiology, Faculty of Medicine, Universiti Kebangsaan Malaysia, Kuala Lumpur, Malaysia; ^3^Department of Medicine, Faculty of Medicine, Universiti Kebangsaan Malaysia, Kuala Lumpur, Malaysia; ^4^Gastroenterology Unit, Faculty of Medicine, Universiti Teknologi MARA, Sungai Buloh, Malaysia

**Keywords:** irritable bowel syndrome, constipation, depression, gut-brain axis, subthreshold depression

## Abstract

Patients suffering from irritable bowel syndrome (IBS) may have some form of affective disorders that may worsen their symptoms. Lack of screening among IBS patients is one of the reasons for depression unawareness among healthcare providers. The present study was conducted to evaluate the prevalence of depression among patients with constipation-predominant IBS (IBS-C). A total of 240 IBS patients who fulfilled Rome III criteria were enrolled. The psychiatric assessment was evaluated using the Center for Epidemiologic Studies Depression Scale Revised (CESD-R). Twenty items in CESD-R scale measured symptoms of depression in nine separate groups. Patients were categorized into five different subgroups: major depressive episode, probable major depressive episode, possible major depressive episode, subthreshold depressive symptoms, and without clinical significance of depression. Out of the 240 patients with IBS-C, the majority (*n* = 161, 67.1%) had no clinical significance of depression. Seventy seven (32.1%) had subthreshold depression and only two (0.83%) patients were regarded as having probable a major depressive episode. No patient was categorized into a major or possible major depressive episode. The prevalence of subthreshold depression was the highest among female (72.3%) patients with 51.1% being single, 44.7% were married, and 4.3% were divorcees. When stratified according to ethnicity, subthreshold depression was highly prevalent among Malays (76.6%), followed by Chinese (19.2%), and Indians (2.1%). A high percentage of the patients were found to be non-smokers (93.6%) and had lower income of less than RM 5000 (USD 1250) per month (89.4%). The moderately high prevalence of subthreshold depression among patients with IBS, justifies psychological evaluation in all patients with functional gastrointestinal disorders.

## Introduction

Constipation-predominant irritable bowel syndrome (IBS-C) is a common functional gastrointestinal disorder with a prevalence ranging from 10 to 16% among multi-ethnic population in Malaysia (Tan et al., 2003; Rajendra and Alahuddin, 2004; Lee et al., 2012). It is one of the subtypes of irritable bowel syndrome (IBS). Several studies verified the psychological and psychosomatic symptoms such as depression, anxiety, insomnia, and headaches that were strongly associated with IBS (Tan et al., 2003; Gibson et al., 2011). A recent meta-analysis study reported depression was higher in all subtypes of IBS compared to control (Lee et al., 2017). Sub-analysis of five separate studies showed that depression was found to be the highest among IBS-C compared to diarrhea-predominant (IBS-D) and mixed-type IBS (IBS-M) (Lee et al., 2017). Cultural habits and language were spotted to be responsible for the differences in symptoms’ interpretation of IBS in the Eastern countries (Chuah and Mahadeva, 2018). Moreover, dietary practices in terms of fiber intake influenced the prevalence of functional constipation between Eastern and Western countries (Chuah and Mahadeva, 2018). The pathophysiology of IBS is complex and remains inadequately understood, but it is most likely due to complex interactions between the nervous system, immune changes and hormonal dysregulation (Nam et al., 2010; Fichna and Storr, 2012; Stasi et al., 2012). Environmental and genetic factors, which include social learning, early life experience, trauma, and social stress, act together bidirectionally with autonomic nervous system and hypothalamic-pituitary-adrenal (HPA) axis (Van Oudenhove et al., 2016). The HPA axis is the core endocrine stress system in humans. It provides an interesting link between the brain and enteric nervous system (de Wied et al., 1993).

Depression is one of the most common mental disorders, worldwide. The prevalence of depression in the general population is approximately 8–12% in Malaysia, regardless of the geographical differences of the study settings. Based on the American Psychiatric Association Diagnostic and Statistical Manual (fifth edition), depression is defined as deterioration of state from previous function with the presence of psychological complaints. It includes dysphoria, as the main complaint with other symptoms such as anhedonia, feeling guilty or worthlessness and recurrent suicidal ideation, together with somatic symptoms. The symptoms include significant weight change or change of appetite, sleep disturbance, abnormal physical movements such as agitation or retardation, feeling fatigue and difficulty to concentrate (First, 2013). Approximately 25.7% of IBS patients were reported to have borderline and clinical significant depression based on the Hospital Anxiety and Depression Scale score (Midenfjord et al., 2019). Using the same tool, the percentage was a bit more (38.6%) between Korean patients with IBS compared to healthy controls. Additionally, they reported that individuals with depression have a negative impact to their quality of life and severity of visceral pain (Cho et al., 2011).

Pharmacological therapy like anti-depressants, that act centrally, may be beneficial to treat psychiatric symptoms that coexist with IBS, as well as visceral hypersensitivity and gastrointestinal motility (Morgan et al., 2005). However, a recent meta-analysis involving 53 randomized controlled trials (RCT) using tricyclic antidepressants, selective serotonin re-uptake inhibitors (SSRI) and placebo demonstrated no improvement in symptoms (Ford et al., 2019). Additionally, the use of various psychological therapies such as hypnotherapy or relaxation therapy failed to improve IBS symptoms (Ford et al., 2019). As an alternative therapy, several theories have been put forward to link between gut dysbiosis, gut-brain axis and IBS. Gut dysbiosis might affect gut-brain axis in IBS leading to psychological symptoms (Tana et al., 2010). Introducing probiotics with an additional polydextrose as a therapeutic approach in IBS patients demonstrated a reduction in colon transit time, stool pH and constipation-related symptoms (Bahrudin et al., 2020).

It is essential to manage IBS that presents with physical symptoms and psychosocial comorbidities. The status of psychiatric problems may vary from impaired interpersonal relationship, social dysfunction, poor quality of life, and suicidal ideation (Spiegel et al., 2007; Zamani et al., 2019). Healthcare practitioners are necessary to be alerted on this issue to ensure early detection of depression among IBS and to plan for appropriate strategies. The aim of the present study was to determine the prevalence of depression among IBS-C patients. We also aimed to investigate if there is any relationship between IBS-C patients with symptoms of depression and the sociodemographic data.

## Materials and Methods

### Participants

This study received ethical approval from Universiti Kebangsaan Malaysia Research Ethics Committee (Reference number: FF-2016-186). All patients were provided with written informed consent forms. Patients were recruited from the Gastroenterology clinic at Universiti Kebangsaan Malaysia Medical Centre from June 2016 until April 2017. The inclusion criteria for this study were individuals aged above 18 years, who satisfied Rome III criteria for the diagnosis of IBS. The exclusion criteria were those with organic gastrointestinal diseases (examples; inflammatory bowel disease and colorectal carcinoma), psychiatric disorder (example: major depressive illness). Subjects must not consumed any pre- or probiotics in less than 2 weeks prior to the recruitment.

We recently published on the use of probiotics in the treatment of IBS-C (Bahrudin et al., 2020). Using the same cohort and additional number of patients, we screened them with this survey. This is a purposive study, whereby from our judgment this is the best way to get a representative sample of IBS patients. A total of 249 patients fulfilled the diagnostic criteria for IBS-C. However, five patients withdrew from the study. Four were defaulters consisted of three failed follow-up and a single person who took different type of probiotics. All nine subjects did not complete the questionnaires. Finally, a total of 240 patients completed the study. Majority of the patients were women (62.9%). In terms of ethnicity, Malays were predominant with 70.5%, followed by Chinese (27.9%), Indians (2%), and others (2%). The median age was 30 years old. Based on the educational level, a higher percentage (60.8%) of patients received tertiary level of education. There was a single subject with no education level. The interview process was guided by the research assistant using layman term and some examples were given especially difficult questions. High education level did not correlate well with high income, whereby only 7% the patients had the highest income of more RM5000 per month (USD 1250). Majority of the patients were non-smokers (97.1%) and never consumed alcohol (99.6%). Only 10% of the recruited patients consumed high fiber diet (more than 5 servings per day). One serving was equivalent to ½ cup of fiber. Pertaining to their daily physical activities, only 12.9% habitually performed daily physical exercise while 33.3% did not actively exercise. Details are shown in Table 1.

**TABLE 1:** Baseline characteristics of 240 constipation-predominant irritable bowel syndrome (IBS-C) patients.

	Total number of patients (*n* = 240)
**Gender, n (%)**	
Male	89(37.1%)
Female	151(62.9%)
**Median age (IQR)**	30 (11)
**Ethnicity, n (%)**	
Malay	169(70.5%)
Chinese	67(27.9%)
Indian	2(0.8%)
Others	2(0.8%)
**Marital status, n (%)**	
Single	116(48.3%)
Married	118(49.2%)
Divorcee	6(2.5%)
**Education level, n (%)**	
Primary	7(2.9%)
Secondary	48(20.0%)
Tertiary	146(60.8%)
Postgraduate	38(15.9%)
None	1(0.4%)
**Monthly income, n (%)**	
<RM 1500	106(44.2%)
RM 1501- 5000	117(48.8%)
>RM 5000	17(7.0%)
**Alcohol intake, n (%)**	
Yes	1(0.4%)
No	239(99.6%)
**Smoker, n (%)**	
Yes	7(2.9%)
No	233(97.1%)
**Fiber intake, n (%)**	
Low	134(55.8%)
Medium	82(34.2%)
High	24(10.0%)
**Physical exercise, n (%)**	
Never	80(33.3%)
Sometimes	129(53.8%)
Habitually	31(12.9%)

*IQR, Inter-quartile range.*

### Measurement and Procedure to Calculate the CESD-R Symptom Score Among IBS Patients

All patients were screened for depression using the 20 items of the Center for Epidemiologic Studies Depression Scale Revised (CESD-R) (Eaton et al., 2012). The scale measures symptoms of depression from nine different groups as defined by the American Psychiatric Association Diagnostic and Statistical Manual (Fifth Edition). The nine symptoms include sadness (dysphoria), anhedonia (loss of interest), change of appetite, sleep disturbance, thinking/concentration disruption, guilt (worthlessness), tired (fatigue), movement (agitation), and suicidal ideation.

The response values for each question were based on the number of days the symptoms occur. A 0 score was given for “not at all” or “less than 1 day,” 1 score for 1–2 days, 2 score for 3–4 days, and 3 score for 5–7 days. The total CESD-R score was calculated as a sum of responses to all 20 questions. The range of possible scores was between 0 (for those who say “not at all or less than 1 day to all 20 questions”) and 60 (for those who say “5–7 days” for all 20 questions). Patients were then categorized into: either having *major depressive episode* [nearly every day for the past 2 weeks has anhedonia or euphoria plus four Diagnostic and Statistical Manual of Mental Disorders (DSM) symptoms groups], *probable major depressive episode* (nearly every day for the past 2 weeks has anhedonia or euphoria plus three DSM symptoms groups), *possible major depressive episode* (nearly every day for the past 2 weeks has anhedonia or euphoria plus two DSM symptoms groups), subthreshold form of depression (CESD-style score of at least 16 but did not meet above criteria) or normal with no clinical significance of depression (CESD-style score less than 16 across all 20 questions).

### Statistical Analysis

Analysis was performed using IBM SPSS Statistics version 23 (IBM Corp., New York, NY, United States) and *p* < 0.05 was considered to be statistically significant. The individual response to each questions in CSED-R was scored manually on the form itself and the final scored entered into the Excel file. Therefore, it was not possible to test for the internal reliability of the questionnaire used.

## Results

### Depression Scale Score Category

A total of 240 patients were analyzed. Minimum score was zero and maximum score was 34. Based on the depression scale category, 67.1% (161 out of 240) of the patients were found to have no clinical significance of depression, with a total score of less than 16. However, 32.1% (67 out of 240) of the patients were grouped as having subthreshold depressive symptoms. Two (0.83%) patients had probable major depressive episode. No patient was categorized into either major depressive episode or possible major depressive episode.

### Subanalysis of Subthreshold Depressive Symptoms

A total of 77 subjects had subthreshold depression. The prevalence was noted to be higher among female (72.3%) as compared to male patients (27.7%). Among them, 51.1% of them were single, followed by married (44.7%) and divorcees (4.3%). The majority was observed among Malays (76.6%), followed by Chinese (19.2%), and Indians (2.1%). They were predominantly non-smokers (93.6%) with lower income of less than RM5000 (USD 1250) per month (89.4%) as seen in Table 1. Eleven out of 77 subjects had underlying medical illnesses. The underlying diseases included: three subjects with hypertension, three with gastritis, two with dyslipidemia and the rest three had all diseases including bronchial asthma, colonic polyps and hemorrhoids.

## Discussion

The existing study aimed to determine the prevalence of psychological disorders among IBS-C subjects. In a total 240 subjects were recruited and met Rome III criteria for IBS-C was included. The other two subtypes IBS-M and IBS-D were excluded. Previous study involving 769 IBS patients, demonstrated 25.7% individuals with psychological distress symptom had severe physical manifestation (GI and non-GI symptoms) and lower quality of life than without psychological symptoms (Cho et al., 2011; Midenfjord et al., 2019). However, this was not always true. Almost half of the psychiatric patients presented late with the gastrointestinal (GI) symptoms. Functional gastrointestinal disorders patients including functional constipation and IBS have higher prevalence of comorbid psychiatric disorders (Stasi et al., 2017). Psychological distress including depression among IBS is not a new finding but studying this in a subtype of IBS, is quite limited. This is the research gap that paved the way for the study to be conducted among a single subtype of IBS and in this case, it was IBS-C. A systematic review and meta-analysis from 73 papers, documented the prevalence of depressive disorder to be 23.3%, and the highest was among IBS-C compared to the control (Zamani et al., 2019). The high prevalence of depression among this group of patients was explained by low responsiveness of 5-hydroxytryptamine (5-HT) secretion in both central and peripheral regions (Lee et al., 2017). Constipation was probably due to low activity of intestinal serotonin system that controls GI motility, as well as low serotonin activity in the central system that controls the mental status, leading to depression (Lee et al., 2017). Our study revealed that nearly one third (>30%) of our study population reported subthreshold depressive symptoms. This finding is vital since subthreshold depression (also known as subclinical depression) is clinically relevant and was found to be highly prevalent (Cuijpers and Smit, 2004). A person below the threshold frequently missed in primary care or community survey. Therefore, it is very important to rigorously evaluate any change in subthreshold depressive symptoms over a period of time. Subthreshold depression could provide an impact and affect the quality of life of patients, increase the utilization of medical services and consequently, would increase the economic burden. It carries a high risk of future major depressive disorder. The increase in the mortality rate was also associated with subthreshold depression (Cuijpers et al., 2007). Hence, intervention for patients with subthreshold depressive symptoms is needed to prevent major depression from occurring.

Another interesting finding that we observed, was a subset of IBS subjects with subthreshold depression had other medical conditions. Generally, depression and anxiety are highly associated with medical conditions such as hypertension (Ginty et al., 2013). A recent publication on systematic review suggested a strong association between IBS and asthma was probably explained by immune system activation and increased intestinal permeability, a link between hyperresponsiveness in atopy and enteric nervous system (Deshmukh et al., 2019). The exact mechanism was not fully understood on how one condition such as IBS leads to another condition such as asthma, or vice versa. Scientists have noticed that the link between the central and enteric nervous system is the gut-brain axis. It is a bidirectional gut-brain communication that connects the emotional and cognitive centers of the brain with peripheral intestinal functions. At the gut level, the intestinal mucosal neuroimmune system reacts to luminal food products, nutrients, bacteria, metabolites, and toxins. Any disturbance to this system may affect cognitive and emotional states as it connects with the central nervous system via the gut-brain axis (Wilhelmsen, 2000). Central nervous system and peripheral tissues have anatomical structures that can mediate stress response. HPA axis is the complex set of core endocrine stress system as it links two important organs; the brain and gut immune system. In addition, the theory of functional cross-talk between gonadal and adrenal gland, that explains several stress-related diseases to be sex-dependent (Habib et al., 2001; Viau, 2002). The principal effectors of the stress response are localized in the paraventricular nucleus of the hypothalamus, the anterior lobe of pituitary gland and the adrenal gland. Corticotrophin-releasing hormone is the primary hypothalamic regulatory peptide, synthesized in the parvocellular cells of the paraventricular nucleus of the hypothalamus. Corticotrophin-releasing factor (CRF) is released into hypophysial portal vessels that access the anterior pituitary gland as a response to stress. CRF binds to its receptor on pituitary corticotrope causing the released of adrenocorticotrophic hormone into systemic circulation. Later, the circulating adrenocorticotrophic hormone acts on the adrenal cortex to stimulate the synthesis and secretion of glucocorticoid from the zona fasciculata. Glucocorticoids may regulate body physiological changes through ubiquitously distributed intracellular receptors (Bamberger et al., 1996). It was hypothesized that psychosocial or physical stress caused an elevation of activity of the HPA and autonomic nervous system leading to overproduction of CRF (Larauche et al., 2011).

A new area of research that fascinates many scientists is the role of microbial dysbiosis and its links with central nervous system causing multiple disorders including IBS (Felice and O’Mahony, 2017). Alteration of gut microbiota was associated with stress related diseases such as IBS and depression (Mayer, 2011; Berrill et al., 2013). Chronic stress itself can lead to changes of gut microbiota by promoting bacterial wall adherence, while the interaction between host and microbiota again can modulate the neuro-immune-endocrine systems (Aguilera et al., 2013). Previous literature reported an elevation of mucosal cell counts especially mast cells among depressed patients (Piche et al., 2008). This has suggested that psychological factors were involved with the process of low-grade inflammatory process in the caecal mucosa of patients suffering from IBS. Chronic stress could modify gastrointestinal motility, HPA axis, autonomic activity, and rectal perception (Chang, 2011). Our recent work on the effectiveness of probiotics in patients with IBS-C created a promising hope for the treatment of stress-related symptoms in IBS patients (Bahrudin et al., 2020).

Supported by all the previous evidence, IBS is a disorder that involves both physical and mental component and should be managed by two disciplines, gastroenterology and neurology. A cross discipline approach is encouraged in order to get a proper management of the disease. Our findings on the subthreshold depression among IBS-C patients is a good start to alert the medical practitioners in this country regarding the importance of having to refer them to the appropriate physicians. The limitation of this study was that we did not recruit all subtypes of IBS due to time constraint. Also, we did not include healthy (non-IBS) subjects for comparison. Future study are warranted in order to explore the mechanisms on the bidirectional influence of gut-brain or brain-gut axis in functional gastrointestinal disease such as IBS.

## Conclusion

Irritable bowel syndrome can lead to poorer quality of life as it can affect the patients’ social and psychological functions. IBS-C is associated with moderately high prevalence of subthreshold depression. It is important for physicians to assess and address psychological symptoms in all patients with functional gastrointestinal disorders. Treatment should be offered to alleviate the psychological symptoms before it proceeds to major depression.

## Data Availability Statement

All datasets generated for this study are included in the article.

## Ethics Statement

The studies involving human participants were reviewed and approved by the Universiti Kebangsaan Malaysia Ethics Committee FF-2016-186. The participants provided their written informed consent to participate in this study.

## Author Contributions

All authors listed have made a substantial, direct and intellectual contribution to the work, and approved it for publication.

## Conflict of Interest

The authors declare that the research was conducted in the absence of any commercial or financial relationships that could be construed as a potential conflict of interest.
